# Comparison of Developers’ and End-Users’ Perspectives About Smoking Cessation Support Through the Crush the Crave App

**DOI:** 10.2196/10750

**Published:** 2019-03-07

**Authors:** Laura Louise Struik, Joan L Bottorff, N Bruce Baskerville, John Oliffe, Susan Crichton

**Affiliations:** 1 Propel Centre for Population Health Impact Faculty of Applied Health Sciences University of Waterloo Waterloo, ON Canada; 2 Institute for Health Living and Chronic Disease Prevention University of British Columbia Kelowna, BC Canada; 3 Propel Centre for Population Health Impact Waterloo, ON Canada; 4 School of Nursing University of British Columbia Vancouver, BC Canada; 5 Innovative Learning Centre Faculty of Education University of British Columbia Kelowna, BC Canada

**Keywords:** mobile app, smoking cessation, tobacco control, young adult, qualitative research

## Abstract

**Background:**

High smoking rates among end-users, combined with their high rates of app use, render this age group as a particularly captive audience for quit smoking apps. There is emerging evidence that apps are an effective way to support smoking cessation among end-users. How the expectations behind the design of apps align with the needs and preferences of end-users, and if this differs by gender, is poorly understood, limiting the ability to evaluate and scale these interventions.

**Objective:**

The objective of this qualitative case study was to detail how the overall design approach of Crush the Crave (CTC), a quit smoking app that targets end-users, compares with young adult women’s and men’s perspectives and experiences, with consideration for the influence of gender.

**Methods:**

Semistructured interviews were conducted with 15 developers involved in the development of CTC and 31 young adult CTC users. Data were analyzed inductively to derive thematic findings of the perceived pros and cons of CTC by both developers and end-users. Findings were grouped under 4 categories (1) technology and platforms utilized for the app, (2) foundation of app content, (3) underlying focus of the app, and (4) look, feel and functionality of the app.

**Results:**

Under the category, technology and platforms utilized for the app, it was found that both developers and end-users agreed that apps aligned with the needs and preferences of young adult smokers. Major limitations with the technology identified by end-users were the frequent “glitches” and requirement for internet or data. For the category, foundation of app content,developers agreed that the strength of CTC was in its strong evidence-base. What mattered to end-users, however, was that the content was packaged positively, focusing on the benefits of quitting versus the consequences of smoking. It was found under the category, underlying focus of the app, that the individually-led focus of the app resonated with both developers and end-users, especially young men. Under the final category, look, feel and functionality of the app, it was found that developers were very positive about the app's aesthetics but end-users thought that the aesthetics incited a negative effect. Also, while end-users found it easy to use, they did not find the app intuitive. Finally, end-users thought that, because the app functions were largely based on a user’s quit date versus their ongoing efforts, this often lent to unmeaningful data.

**Conclusions:**

The current study findings highlight the importance of understanding multiple perspectives of stakeholders involved in a mobile-based intervention. By gathering the viewpoints of developers and end-users, both problematic and effective approaches that underlie development goals were revealed as a means of informing the development, implementation, and evaluation of future electronic health (eHealth) interventions.

## Introduction

Smoking remains the number one public health concern, especially among young adults because they continue to maintain the highest smoking rates [1]. Despite comprehensive evidence that the younger a smoker quits (before 34 years of age), the greater the health benefits, a significant number of smokers don’t successfully quit until after the age of 45 [2,3], when the adverse health impacts of smoking are not completely reversible. This is due, in part, to low uptake of cessation interventions by the young adult population [4-6]. This evidence underscores the urgent need to reach young adults with effective smoking cessation interventions, and mobile phone apps present as an attractive means to do so, with young adults representing a particularly receptive audience for quit smoking apps [7,8].

The benefits of mobile phone apps over traditional cessation interventions include the ability to access support anytime, anywhere, via complex functions, including interactive self-monitoring activities, diverse multimedia, and tailored support via context sensors [9,10]. As a result, the quit smoking app market has exploded, with hundreds (over 500) of smoking cessation apps now available on the Apple and Android platforms [11,12]. Despite the plethora of quit smoking apps, few are evidence-informed, theoretically-based, or include the perspectives of end-users [13-15]. Regardless, consumers are downloading these apps to help them quit smoking, with popularity especially high among the young adult population [8]. While emerging evidence indicates that apps may be an effective way to assist individuals with quitting smoking [16-20], and young adults in particular [18], little is known about which aspects of these apps are well designed and address the needs and preferences of young adults.

Researchers have expressed concern about how little detail is provided in relation to the underlying principles of development and design processes of mobile health (mHealth) smoking cessation interventions [10,21,22]. Tomlinson and colleagues [21] have described the current influx of mHealth interventions as a wave of “black boxes” because there is a lack of research detailing the developmental processes of and subsequent expectations for these interventions. The paucity of research on the development of these initiatives reflects a primary concern for health outcomes in the mHealth intervention literature despite the fact that the processes of development are essential to the establishment of optimal and scalable interventions [21].

In addition to the lack of research in relation to the design and development of cessation apps, there is also a lack of research on end-user (those who actually use the apps) perspectives and experiences and if they align with the expectations of development. While some studies have harnessed end-user input during the development of an app [23], the actual experiences of end-users after app roll-out has yet to be interrogated. We don’t know if users’ actual experiences align with the design expectations of the developers. This is a critical step toward enhancing uptake and engagement with interventions. For example, while Ploderer and colleagues [24] did not make explicit the underlying development principles of the DistractMe app, they did describe which end-user experiences aligned with the design intentions of the app. As a result, the authors were able to draw conclusions about which aspects of the app worked well and how development practices can be improved to optimize its effectiveness.

Furthermore, it is well established in the literature that there are significant gender influences on smoking behavior, and that gender-sensitive approaches to quitting smoking have been found to positively influence receptivity to and use of the interventions, as well as mobilize smoking cessation [25-27]. Yet, no efforts to investigate the influence of gender and ways to incorporate a gender-sensitive approach into mobile-based smoking cessation interventions specifically have been found. Because the ways in which young women and men use and benefit from smoking cessation interventions as they engage in quitting smoking may differ, attention should be paid to potential gender-related influences in the ways that an app is developed, as well as how women and men perceive and experience these interventions.

To our knowledge, no studies to date have explicitly aligned developers’ perspectives and end-user perspectives on using an app for cessation. Juxtaposing the processes of development and design that underpin an app against user perspectives and experiences is essential to exposing misalignments and optimizing design practices. The aim of this study was to address an important knowledge gap by comparing the overall design approach of Crush the Crave (CTC), a quit smoking app that targets young adults motivated to quit smoking, with young adult women’s and men’s perspectives. As described in Baskerville and colleagues [23], the features and functions in the app are underpinned by principles of persuasive technology for behavior change and the US Clinical Practice Guidelines on what works to support quitting smoking. This study specifically compares the pros and cons of the app as perceived by the developers of CTC with the perceptions and experiences of young adult smokers who have used the app.

## Methods

### Design

The CTC app was being evaluated through a randomized controlled trial (RCT) [28] at the time of the current study. In the RCT, baseline, 3-month, and 6-month follow-up surveys were collected to assess the effect of CTC on smoking cessation compared to the quit guide. The present study served as a companion study to this RCT. Young adult participants for the present study were drawn from the individuals in the intervention group that completed all 3 surveys (n=307).

Through a qualitative case study design [29], the processes of development that underpin CTC were juxtaposed with end-users’ perspectives and experiences in using the app, a knowledge area that we know little about. A qualitative case study is ideal for engaging in a holistic investigation of a phenomenon on which little is known [29]. Congruent with this methodology, data collection and analysis were qualitative in nature.

Ethics approval for this study was obtained from the University of British Columbia (Okanagan campus) Behavioral Research Ethics Board (Certificate H15-00466).

### Participants

#### Developers

Using a purposive sampling strategy, developers were derived from those who were directly involved in the decision making in relation to the development, design, and implementation of CTC. Developers were put in touch with the principal investigator via an introductory email sent by the senior scientist of CTC and subsequently recruited via email. Altogether, 15 developers provided informed consent and participated in the study. Table 1 provides a description of the key informant sample.

Using a purposive sampling strategy, young adults (ages 19-29) who had been assigned to the RCT intervention group and were either recent quits or still smoking were recruited into the current study. Participants recruited were those who completed the 6-month questionnaire in the RCT and selected “yes” to receiving information about this qualitative companion study. These participants provided their contact information (email and phone number) and, therefore, recruitment was conducted via email, phone calls, and texting. Altogether, 31 young adult end-users provided informed consent and participated in this study. Table 2 provides demographic and smoking behavior data for young adult participants.

### Data Collection

Semistructured interviews were held with developers and end-users, which are less directive and more open-ended [29] to ensure that the perspectives of each sample were captured. Except for one Skype interview with a developer, all interviews were conducted via telephone. Interview questions for developers focused on perceived strengths and limitations in the design of the app, while interview questions for end-users focused on their likes and dislikes related to app design. These conversations were about the overall features and functions of the app rather than the specific content (eg, for developers: “In your opinion, what are some of the best things about the app?” and for end-users: “Looking back over the time you have used the app, what were some of the things you liked best about using CTC?”).

All interviews were audio-recorded and lasted between 30 and 80 minutes. Young adult participants received an honorarium (Can $50/interview) to acknowledge time spent on the study.

**Table 1:** Key informant sample.

Job role		Time of involvement in Crush the Crave development	Involvement with the app
**Men (n=8)**			
	Academic	During	Design
	Academic	During	Design
	Academic	During and after	Design and evaluation
	Clinician scientist	During	Design
	Data systems specialist	During	Branding
	Media developer	During and after	Design and marketing
	Academic	During and after	Design and evaluation
	Senior scientist	During and after	Design and evaluation
**Women (n=7)**			
	First project manager	During	Study management
	Second project manager	After	Study management
	Academic	After	Evaluation
	Academic	During	Literature review
	Academic	After	Evaluation
	Partner organization	During	Design and marketing
	Research coordinator	During and after	Management of social media

**Table 2:** Young adult study population (N=31).

Characteristics		Value
Age (years), mean (SD), range		24.7 (2.7), 20-29
**Gender, n (%)**		
	Female	13 (41.9)
	Male	18 (58.1)
**Education, n (%)**		
	<High school	3 (9.7)
	High school	4 (12.9)
	Some postsecondary	9 (29.0)
	Trade	1 (3.2)
	College	11 (35.5)
	University degree	3 (9.7)
**Income (Can $), n (%)**		
	<15,000	4 (12.9)
	15,000-29,000	4 (12.9)
	30,000-44,999	4 (12.9)
	45,000-59,999	5 (16.1)
	60,000-79,999	3 (9.7)
	≥80,000	3 (9.7)
	Don’t know/refused	8 (25.8)
**Population group, n (%)**		
	Aboriginal	3 (9.7)
	White	22 (70.9)
	South Asian	2 (6.5)
	Other	4 (12.9)
**Home province, n (%)**		
	British Columbia	2 (6.5)
	Alberta	3 (9.7)
	Saskatchewan	4 (12.9)
	Ontario	15 (48.3)
	Quebec	1 (3.2)
	New Brunswick	2 (6.5)
	Nova Scotia	3 (9.7)
	Newfoundland	1 (3.2)
**Smoking status at 6 months, n (%)**		
	Quit smoking	7 (22.6)
	Currently smoking	24 (77.4)

### Data Analysis

Data analysis was conducted simultaneously with data collection, with a minimum requirement of six interviews for saturation [30]. Saturation was ultimately driven by saturation of themes within the overall framework of the study. All interviews were transcribed verbatim. Transcripts and documents were uploaded onto NVivo(QSR International (Americas) Inc.). An analysis was then guided by the framework approach, which consisted of a series of interconnected stages (familiarization, identifying an analytic framework, indexing, charting, and mapping and interpretation), enabling a coherent and transparent account of the analysis [31].

Data from interviews with young women were kept as a separate dataset from those with young men to enable the lead author to compare and contrast young women’s and men’s experiences and identify notable gender-related influences in the datasets and findings. After the first four interviews with developers, young women, and young men, a coding framework was developed, identifying key themes in relation to their overall perspectives on the app. The thematic frameworks for each dataset were then reviewed and approved by all authors. The frameworks were subsequently used to code the remaining transcripts and revised as added to as new data emerged. The frameworks for young women and men were compared and then combined with important similarities and differences noted. The final frameworks were then transferred to tables. Representative quotes were selected to illustrate key themes and subthemes.

## Results

The perceived pros and cons of CTC fell into 4 categories: (1) technology and platforms utilized for the intervention (relates to perspectives on delivering a cessation intervention via a mobile app, with the additional support of social media, (2) foundation of app content (relates to perspectives on the principles that underpin the app design), (3) underlying focus of the app (relates to perspectives on the implied focus of the app design based on the technology used and the dominant features and functions that were built into the app), and (4) look, feel and functionality of the app (relates to perspectives on the overall design of the app and how it is packaged for young adults).

### Technology and Platforms Utilized in the Intervention

Developers described the use of mobile technology as a natural evolution of tobacco control efforts—that it was necessary to keep up with current trends of using digital media in tobacco control, and health care more broadly. Along with this vein, there was often a sense of urgency to take tobacco control efforts into the realm of electronic health (eHealth). Notably, this urgency was underpinned by a desire to get it right and to not just put something out there that wasn’t given much thought. It was clear that the developers were invested in designing an eHealth approach that would work rather than something that was simply novel:

This is going to be the app or some version of something like that—some portable, accessible, customizable, personalized thing. Every trend is going to that; this is the future…We need to understand how to get this right because if we don’t, the tobacco companies will and other people will, and we [will be] competing against all kinds of other things. If we can get this right, this [will become] a frontline for public health…If we don’t go there, we’re losing an enormous opportunity to make a big difference in people’s lives.

Informant #9, male

Both developers and end-users thought that a cessation app has an edge on other cessation intervention formats because an app would meet diverse populations of young adults where they are at, both in terms of being ready at hand and in terms of the type of support they’d want to receive. For example, a key informant reflected on how young adult smokers have been neglected in relation to tobacco control efforts, mainly because the health-seeking behaviors of young adults are diverse and can differ from their older counterparts, with whom young adults are often grouped together in cessation initiatives (eg, going to a physician, counseling). She described CTC as an intervention that was designed to align with how young adults seek health information and support, saying that CTC “is a place that they can go to get that kind of support that is not totally out of their comfort zone”. End-users agreed that the use of mobile technologies for a smoking cessation intervention fit with the needs and preferences of their age group. They particularly described the portability of CTC, since it is delivered via an app, as one of the most liked aspects of using the CTC app:

Just the fact that it is an app and I can bring it with me instead of like having a chart at home or something like that where it’s not very portable. The app you can bring with you wherever you go ‘cause chances are you’re gonna have your phone on you.

Participant #2, female smoker

Developers and end-users agreed that integrating the app with social media platforms enabled easier access and opened opportunities to reach young adults with cessation support. While end-users did not perceive any downfalls to delivering cessation support via an app, developers were concerned about the ever-changing nature of technologies and platforms, and user preferences and implications this had for keeping interventions relevant:

Things go by the wayside so fast that by the time we probably tried to work something out, Snapchat would be no longer the thing that young adults are using. They would be on to the next thing. So, it’s so hard to keep up with technology just because it’s such a quick pace nowadays. Like phones—new phones come out every six months now…similar to apps and similar to the different platforms that people are using.

Informant 11, male

While developers didn’t anticipate technical problems, this was a frequent problem encountered by end-users (eg, freezing), leaving them feeling empty-handed in trying to get help with quitting smoking. These glitches frequently drove users away from the app. A few end-users also lamented the fact that the app could only be used if they had an internet or data connection. This often resulted in limited use of the app, as well as disappointment in not being able to access the app and its features during times that they needed it (eg, during a craving).

### Foundation of App Content

The most talked about strength underlying the design of the app among developers was that its content and development was informed by evidence and theory. In particular, developers described the inclusion of end-users via focus groups during the development of the design concept, as well as to test the app prototype, as a key strength. This led many developers to suggest that the app would be relevant to various subpopulations of young adults, and it was often described as a “one-stop shop” or a “Cadillac” design because it included so many evidence-informed features. As demonstrated in the statement below, developers thought that the evidence-informed nature of the app gave CTC an edge on the growing number of quit smoking apps available:

There were plenty of stop smoking apps out there but they weren't particularly good ones. They weren’t…[based] on the evidence of what we know works, and they weren't based on good theory around behavior change…There was an obvious gap there and we wanted to try and make sure there was something good available for young people who would be looking for apps.

Informant #14, male

A few developers, however, had counterviews about the staying power and transferability of clinical practice guidelines and long-standing theories related to behavior change in the eHealth context. Developers were cautious about taking guidelines that were designed for a clinician in a clinic and putting them into an app for users to support self-management of health behavior. Some developers were also concerned that the app was built upon the notion of a successful quit trajectory. Rather than making room for the relapses that frequently occur during quitting, the app implies that quitting is straightforward (eg, that end-users can expect to be smoke-free by the time they reach their quit date). A challenge during development was balancing an idealized notion of quitting with the reality of a quitting trajectory:

The biggest limitation that I see is that they [the features of the app] are based on a generalized model of quitting smoking, even if you think about the theoretical model, it just kind of goes forward right? And they will acknowledge that yes people go back and forth…[But] the reason you don’t draw…anything other than an arrow, aside from maybe something going backward occasionally, is that it looks like spaghetti when you actually think about how people change...It’s a complete mess...people go forward, backward, sideways...But that’s almost impossible to put on a program. And so, we run this risk of creating a bit of a fiction, an evidence-informed fiction, if we may.

Informant #9, male

When asked about how gender was considered during the development of the app, key informants explained that the app was designed to be “gender neutral” and therefore, was not underpinned by consideration for gender. Some key informants thought that this led to them defaulting to a rather male-centric app:

Definitely, the app is very much branded to your white male. Especially with the default images at the front with the rock climbing and that sort of thing so—that initial white male [feel] is unfortunately very prominent when you look at it through that lens.

Informant #10, female

What end-users liked about the foundation of the app content was that, despite it being developed and informed by evidence and scientific institutions, the content was delivered in a fun and positive way. Many end-users described how they typically expect to receive messages about the negative consequence of smoking versus the positives of quitting. Because the app focused on the latter, end-users were more receptive to it. One young woman who still smoked provided a detailed description of why the ways in which the content was delivered was appealing to those her age:

[The app] is not just pure scientific, “this is what you have to do, this is why you need to quit smoking, these are all the chemicals that are in it,” you know what I mean? There was more of a fun aspect to it. Where it seemed like it wasn't so serious…And so I liked that about it, right? 'Cause it wasn't so stuffy, it wasn't so clinical. It was more like, “okay, we know that you wanna quit, but we're not gonna judge you too severely if you don’t,” right? I don’t wanna say that it didn’t seem as serious, it just didn’t seem as clinical.

Participant #22, female smoker

### The Underlying Focus of the App

A strength of the underlying focus in the design of CTC according to both developers and end-users was the individually-led nature of the intervention. In this respect, the CTC app was designed to enable end-users to quit on their own and track and modify their smoking behaviors without consultation with others, health professionals and personal networks alike. It was agreed by both developers and end-users that this individually-led focus was compatible with how young adults generally approach quitting smoking, which is on their own. In particular, this design feature was viewed as a strength for reaching young men. One female key informant described how the self-driven nature of the app played to many men’s preferences for self-management when it comes to their health:

I mean, men like tools…it’s like a tool, it’s a do it yourself, I don’t have to tell anybody, I don’t have to ask anyone [thing]…Yeah, I think the fact that it’s a do it myself; [I don’t have to] ask for help sort of thing, and it’s like one-stop shop.

Informant #5, female

In keeping with these sentiments, young men were particularly vocal about liking the self-led design of CTC, explaining that they are very private and like to take on quitting smoking independently. Young men frequently stated that they did not like to share personal things, such as the decision to quit smoking, with anyone—friends and family alike. Young women, on the other hand, were more inclined to draw on social support options within the app (eg, the quit buddy).

An aspect that women liked, but men were not keen about, was the “give and take” focus in the app. While men wanted to be less engaged with data entry, women appreciated the need to be engaged throughout the process because it provided them with opportunities to reflect on their smoking and to develop new ways of coping:

Yeah, so it’s not just like documenting data, it’s giving you something to do…And, it’s like—it also takes and gives to you… it’s like, “what are you doing when you crave or when you do have a cigarette…what you’re feeling then,”…Some of the other apps, most of them, you have to buy that information. Like you have to pay, God only knows how much, and this app, it’s just like, okay it’s there for you.

Participant #12, female, smoker

### Look, Feel, and Functionality of the App

In relation to the app’s aesthetics, developers thought that they were done well:

There is something that has to catch their eye, and we talked a lot about the design of it—like literally the design, like the logo, that sort of stuff because that sort of stuff does matter...It’s a nice logo, it’s different, it’s a cool name…I think this team got it.

Informant #9, male

Interestingly, almost all end-users, women and men alike, did not like the way the app was aesthetically packaged. In keeping with the one key informant’s thoughts, they described the app as too dark, espousing a negative effect, which contradicts the otherwise positive orientation of the app. This is captured in the following statement:

One thing that I did notice is that the background colors; they’re a little dreary. Like the black and the orange and like when you first click on it….it could be a little more like brighter and happier….A different color scheme I think would work a lot better…And like black, from a psychological standpoint, black and red, they’re like angry negative colors…So if you put more like blues and greens and like yellows and like summer colors and things like that in it, it might change people’s you know mood a little bit more, psychologically without them even knowing.

Participant #2, female smoker

One thing that end-users stated that they liked about the look and feel of the app was that it was easy to use. This is demonstrated in the following quote by a young woman:

It was easy to get to, easy to use. Especially like being a mom…it was easy and simple. It wasn’t overly complicated—like to start, like the start-up was [easy to] enter stuff…it wasn’t overly long…[My son] only lets me use my phone for like two seconds at a time.

Participant #25, female smoker

Despite that the app was often described as easy to use, end-users agreed that navigating the app was not very intuitive. They often questioned why there were so many subpages of pages, which led to “hidden” features they were unaware of, or found out about late in their quit smoking journey. They also expressed frustration with being sent out of the app to access certain tools (eg, craving distractions).

A limitation identified by end-users in relation to the functionality of the app was that the functions (awards, leaderboard, health calculators) were based on a point in time—the user’s quit date. One young woman described how the calculators (money saved, health regained) could be very powerful for quitting smoking but that the glitches in the app prevented her from logging her smoking and cravings, which lent to inaccurate statistics displayed on the app:

I couldn’t actually log how many cigarettes and stuff I had …[so] it’s not accurate. But if it was accurate it [would be] cool to see like, you know, money saved, like, oh hey, I saved $100 smoking so far. Like you know, it’s something to be proud of.

Participant #30, female smoker

## Discussion

### Principal Findings and Implications

To date, there has been a primary reliance on quantitative research evidence for evaluating eHealth interventions. The rich findings as a result of harnessing perspectives of both developers involved in app design and end-users using CTC hold potential for contextualizing why certain aspects of the app worked well and others did not work well, and in doing so extend the results of the quantitatively focused evaluations of CTC [28]. The importance of gaining knowledge about the implementation processes and experiences associated with interventions has been recognized [32-34] and is supported by the findings in the present study.

This qualitative study is novel in that it provides a formal comparison of the developers’ and end-users’ perspectives providing much needed empirical evidence to the eHealth literature. By gathering the viewpoints of developers, both problematic and effective approaches that underlie development goals were revealed. Indeed, developers and end-users in the current study findings can advance the development and implementation of eHealth interventions, holding great promise to improve their uptake and impact compared to their current overall status, which is often poor or undecided [35-37].

The positive feedback by both sample groups on entering the mobile market for supporting smoking cessation demonstrates that the use of mobile phone technology is a much-needed and relevant approach for supporting quitting smoking among young adults. Emerging evidence of the efficacy of some evidence-informed apps for quitting smoking [11,18,19,38] confirms that smoking cessation interventions are appropriately positioned in the mobile context. In the recent RCT of CTC (Baskerville et al in press), it was found that, while CTC was not superior to the control condition, the prolonged abstinent rate and thirty-day abstinent rate for CTC was comparable to other research on smoking cessation smartphone apps [11,38]. The widespread support by end-users in this study confirms support for entering the mobile space to reach young adult smokers specifically.

While it is established that we are on the right track with using mobile technology, the differential and sometimes problematic experiences with CTC among end-users bring forward questions about how and when the target end-users are appropriately engaged in intervention research. Although CTC was designed and developed with input from young adult focus groups, the findings of the study revealed gaps between the developers’ perspectives and the perspectives and experiences of the end-users. As previously described [23], apart from one pilot test run with end-users, engagement with end-users primarily consisted of preintervention focus groups. This raises questions about the value in relying primarily on preintervention focus groups in the development of mHealth interventions. Recent research has detailed the problematic position of end-users in the development of eHealth interventions—they are often peripheral stakeholders that have marginal engagement during the development [37], which was the case for CTC. Others have argued that positioning end-users in this way have the potential to lead to a mismatch between technologies and end-users’ daily lives, habits, and rituals [37], lending to usability problems and high attrition rates [35,39,40]. The findings of this study provide further support for these concerns. For example, many end-users complained about technology glitches (eg, freezing) and the lack of intuitive design (eg, features and functions were not easily accessed or located). Users often cited these issues as contributing to their disinterest and eventual disengagement with the app.

Researchers in eHealth have highlighted the need for and benefits of harnessing end-user perspectives during the design and development of eHealth behavior interventions. For example, involving end-users has been shown to improve usability [41], prevents the inclusion of superfluous features [42], and can be more economical in that money is not put into bad design aspects [41]. However, what is lacking in the literature is how and when to engage end-users in eHealth intervention research. This raises questions about the appropriateness and effectiveness of the ways in which end-users were engaged during the development of the app. Recently, eHealth researchers have begun to pay close attention to the developmental requirements of health behavior interventions so that these interventions can be more effectively developed and subsequently scaled up. In this vein, it has been suggested that multiple formative evaluations be conducted with end-users to test design assumptions and prototypes [37,43]. One way to address this need would be the inclusion of end-users on the development team in addition to conducting feature-level analyses based on log data (eg, google analytics), such as that used by Heffner and colleagues [44]. These practical strategies may help address the need for more comprehensive and frequent end-user input, while also addressing issues in relation to time, resources and funding that are commonly associated with evaluating eHealth interventions.

A recently published qualitative investigation of CTC found that end-users did not engage with the social support aspects of CTC due to fear of judgment, failure in quitting, and shame in smoking [16]. Considering the current study finding in relation to end-users’ preference for an individually-led intervention, it can be reasonably argued that the social support features were not designed in a way that aligned with the nature of the intervention. This highlights the need to pay attention to how content is presented and if it aligns with the overall focus of the intervention (eg, an app-based forum versus a public social media page).

Furthermore, while developers focused on the scientific background of the app content, end-users focused, again, on how this content was presented. Positive framing of the app and its content appeared to play an important role in uptake and use of the app. Despite some developers concerns about uptake, end-users described how the apps focus on the benefits of quitting versus the consequences of smoking largely influenced their desire to download and use the app. Given traditional approaches that frequently played on fear (eg, pictures of negative health consequences on cigarette packs), guilt (eg, neonatal health consequences), or judgment [45], end-users welcomed the positive and encouraging nature of the app. In the debate between positive and negative framing of content for tobacco control efforts, the findings of this study extend existing evidence that positive message framing resonates with smokers [45,46] and specifically resonates with the young adult population [47].

CTC was designed to be “gender-neutral.” A gender-neutral approach in cessation interventions can be understood as gender-blind, running counter to best practice frameworks and guidelines for treating tobacco dependence [48,49]. Researchers have raised concerns about the lack of attention to gender in cessation interventions given evidence that gender-related factors play a significant role in tobacco use [50,51]. For example, men have a long history with tobacco use and dependence that has been linked to masculinities and gender roles. Similarly, gender-related factors have been implicated in women’s smoking, with femininities and attractiveness associated with women’s smoking and gendered factors such as concern for weight gain contributing to smoking maintenance [52]. Furthermore, a gender-neutral approach puts emphasis on the end-goal (quitting among end-users), ignoring gender-related factors that may limit ones’ ability to quit smoking. For example, oftentimes, young women have been reported to take up and maintain smoking/substance use to cope with current trauma or past trauma (eg, domestic violence) [53]. Given reports that 1 in 3 women experience trauma [54], a gender-neutral approach fails to account for and address this issue. Along with this vein, discourses in relation to gender roles and norms (eg, women are responsible for their personal and familial health) may be reinforced through a gender-neutral approach because gender-related diversity and differences are ignored and unaddressed. It is naïve to focus on the end-goal (in this case, smoking cessation) and not account for established factors that may prevent one from achieving important health behavior changes, like quitting smoking. Indeed, in keeping with the positive impacts of gender-sensitive interventions [25-27,55], it is strongly recommended that mobile-based smoking cessation interventions be designed to address gender-related factors influencing smoking and quit efforts.

That end-users did not like the aesthetics of the app because it stimulated negative emotions, brings attention to the emotional side of end-user experiences, something that has been neglected by researchers investigating human-technology interactions [56]. Thuring and Mahlke [56] assert that researchers are primarily focused on effectiveness, efficiency, and satisfaction at the neglect of other aspects, such as the aesthetics of system design and emotional experiences during system usage. In addition, researchers have found that the visual attractiveness of an app also influences perceived usability [56,57]. It is urged, therefore, that future development practices in the area of mHealth foreground consideration of the aesthetics of apps alongside effectiveness and efficiency.

### Limitations

There are several limitations to this study. As with all self-report research, there may be perspectives and experiences that were not captured during the interviews. Also, interviews with developers were conducted a couple of years after the app was developed between 2012 and 2014. In this regard, perspectives of developers may have been influenced by current advancements in technologies, as well as their knowledge of what aspects of the app worked well and which ones did not work well. In the same way, some end-users were interviewed up to a year after they entered the RCT study, potentially limiting their ability to recall their experiences. To address this potential limitation, reflective questions were posed during interviews with both samples to assist participants in recalling events and experiences, and when necessary follow-up questions and probes were used to capture additional details. Also, the young adult sample largely consisted of those who continued to smoke and were primarily Caucasian, thus affecting the generalizability of the findings. Finally, evolutions in technology inherently challenge the transferability of eHealth research.

### Conclusion

Given the rich findings of this study, particularly in relation to some of the stark differences found between developers and end-users, the inclusion of multiple perspectives is a much-needed addition to the eHealth literature. By gathering the viewpoints of developers, both problematic and effective approaches that underlie development goals were revealed. Incorporating a gender-based lens in this study brought forward nuances between young women’s and men’s perspectives and experiences with the app. Indeed, by harnessing data from both developers and end-users, the current study findings can advance the development and implementation of eHealth interventions.

## Abbreviations

CTC: Crush the Crave
eHealth: electronic health
mHealth: mobile health
RCT: randomized controlled trial
